# Supplementary material for: A Streptomyces sp. NEAU-HV9: Isolation, Identification, and Potential as a Biocontrol Agent against Ralstonia solanacearum of Tomato Plants
Source: Microorganisms. 2020 Mar 1;8(3):351. doi: 10.3390/microorganisms8030351 (PMC7142955; doi:10.3390/microorganisms8030351)
Supplement: Supplementary file 1 [file microorganisms-08-00351-s001.pdf]

A *Streptomyces* sp. NEAU-HV9: Isolation, identification, and potential as a biocontrol agent against *Ralstonia Solanacearum* of tomato plants

Ling Ling[1], Xiaoyang Han[1], Xiao Li[1], Xue Zhang[1], Han Wang[1], Lida Zhang[1], Peng Cao[1], Yutong Wu[1], Xiangjing Wang[1], Junwei Zhao[1,*] and Wensheng Xiang[1,2,*]

1 Key Laboratory of Agricultural Microbiology of Heilongjiang Province, Northeast Agricultural University, No. 59 Mucai Street, Xiangfang District, Harbin 150030, China; LLYNL2621161093@163.com (L.L.); hanxy139251@163.com (X.H.); Lx1244070003@126.com (X.L.); zhangxue_968425@163.com (X.Z.); wanghan507555536@gmail.com (H.W.); yone910310@163.com (L.Z.); cp511@126.com (P.C.); 18103699151@163.com (Y.W.); wangneau2013@163.com (X.W.)

2 State Key Laboratory for Biology of Plant Diseases and Insect Pests, Institute of Plant Protection, Chinese Academy of Agricultural Sciences, Beijing 100193, China

* Correspondence: guyan2080@126.com (J.Z.), xiangwensheng@neau.edu.cn (W.X.)

Table S1. Growth and cultural characteristics of strain NEAU-HV9 after 2 weeks at 28 °C

Medium	Growth	Aerial mycelium	Substrate mycelium	Diffusible pigment
ISP 1	Good	White	Moderate Yellow	Vivid Yellow
ISP 2	Good	White	Brilliant Orange Yellow	Light Orange Yellow
ISP 3	Good	White	Brilliant Orange Yellow	Light Yellow
ISP 4	Good	White	Brilliant Yellow	Dark Greenish Yellow
ISP 5	Good	Moderate Yellow	Grayish Yellow	Moderate Yellow
ISP 6	Good	None	Moderate Yellow	Moderate Yellow
ISP 7	Good	Pale Greenish Yellow	Dark Olive	Moderate Olive
NA	Good	None	Moderate Yellow	Moderate Greenish Yellow
BA	Good	None	Brilliant Orange Yellow	Light Orange Yellow
CA	Poor	White	Pinkish White	None

Table S2. Physiological and biochemical characteristics of strain NEAU-HV9, Abbreviation: +, positive; –, negative.

Characteristic	Result	Characteristic	Result
Growth at/with:		Decomposition of	
Temperature range (°C)	15-37	Adenine	+
Growth pH range	5-9	Casein	−
Maximum NaCl	7	Hypoxanthine	+
Production of H_2S	+	Tyrosine	+
Reduction of nitrate	−	Xanthine	+
Coagulation and peptonization of milk	−	Hydrolysis of	
		Aesculin	+
Liquefaction of gelatin	−	Starch	+
Carbon source utilization		Nitrogen source utilization	
L-Arabinose	−	L-Alanine	+
Dulcitol	−	D-Arginine	+
D-Fructose	−	L-Asparagine	+
D-Galactose	+	L-Aspartic acid	+
D-Glucose	+	Creatine	−
Inositol	+	L-Glutamic acid	+
Lactose	−	L-Glutamine	+
D-Maltose	+	Glycine	+
D-Mannose	+	L-Proline	+
L-Raffinose	+	L-Serine	+
D-Ribose	−	L-Threonine	+
D-Sorbitol	−	L-Tyrosine	+
D-Sucrose	+		
D-Xylose	−		

All data was obtained from this study.

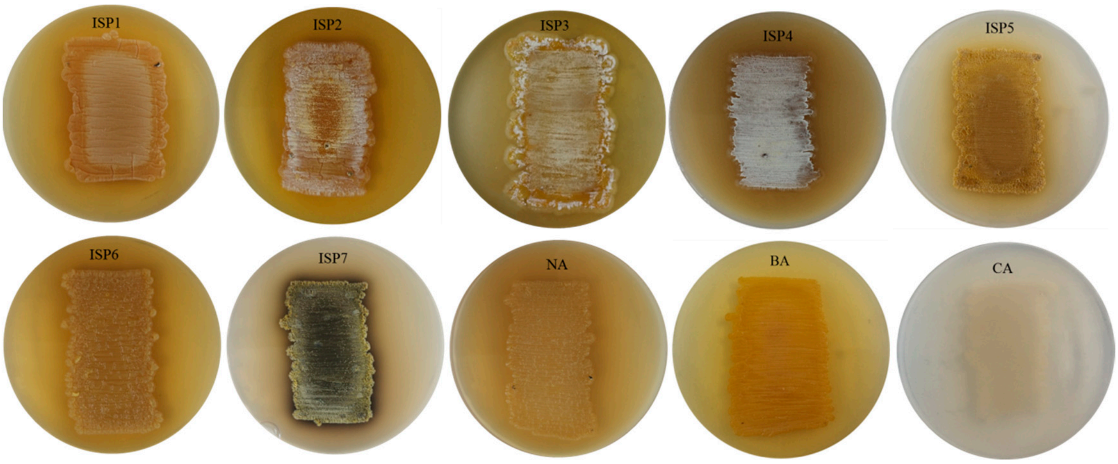

Figure S1. Cultural characteristics of strain NEAU-HV9 observed on ISP 1, ISP 2, ISP 3, ISP 4, ISP 5, ISP6, ISP7, Nutrient agar, Bennett's agar and Czapek's agar after being incubated at 28 °C for 2 weeks.

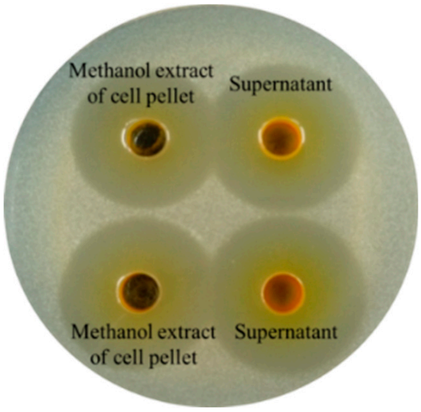

Figure S2. Bioactivities of the supernatant and cell pellet of NEAU-HV9 against *R. solanacearum*.

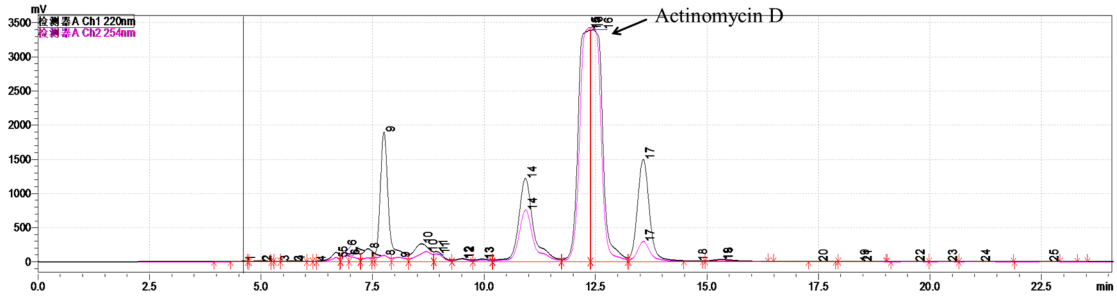

Figure S3. The HPLC profiles of crude extract produced by *Streptomyces* NEAU-HV9.

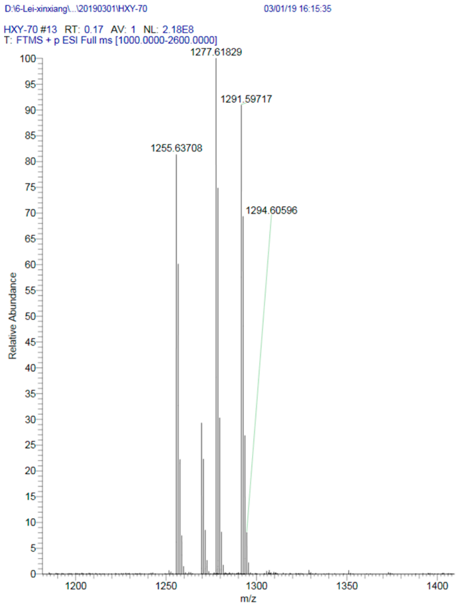

Figure S4. Mass Spectrometry of actinomycin D ($C_{62}H_{86}N_{12}O_{16}Na$: 1277.6).

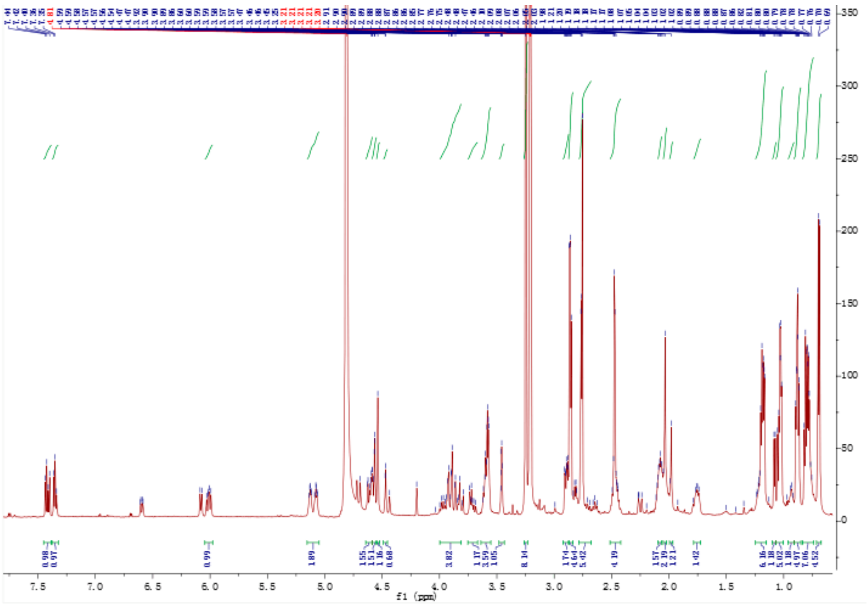

Figure S5. ^{1}H NMR of actinomycin D.

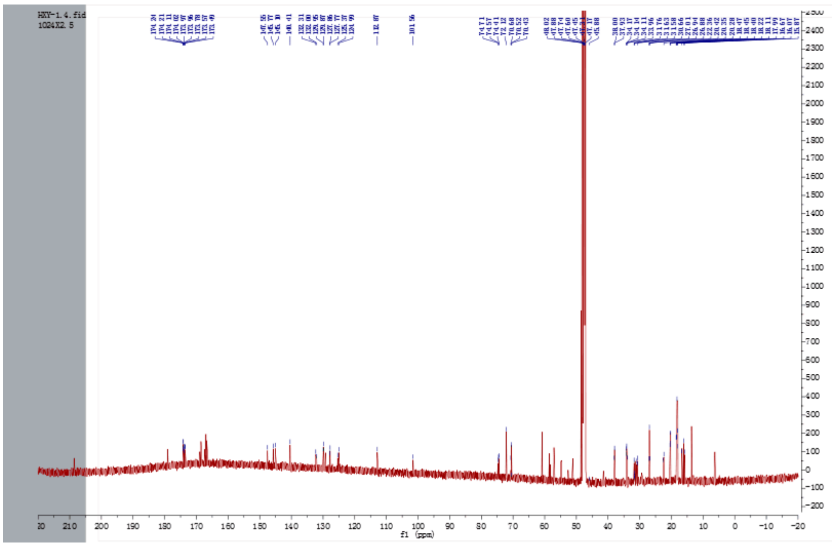

Figure S6. ^{13}C NMR of actinomycin D.

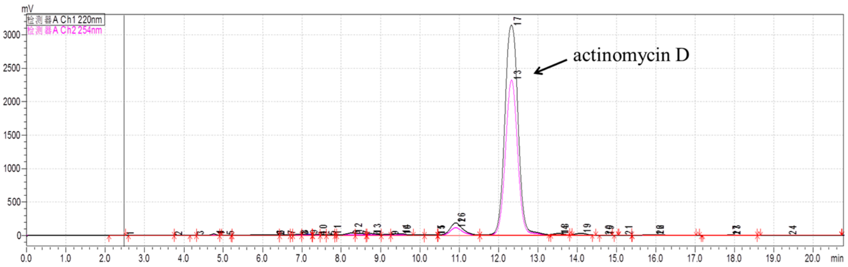

Figure S7. The HPLC profiles of commercial actinomycin D.

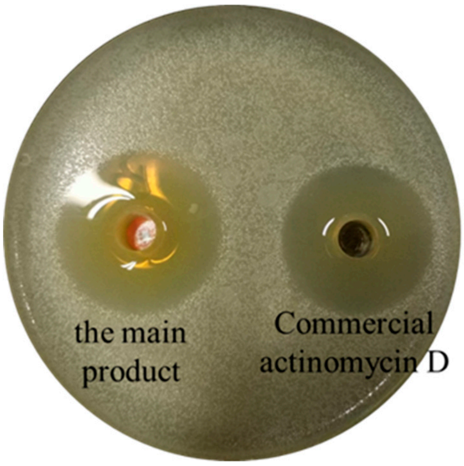

Figure S8. Bioactivities of commercial actinomycin D and the main product of NEAU-HV9 against *R. solanacearum*.

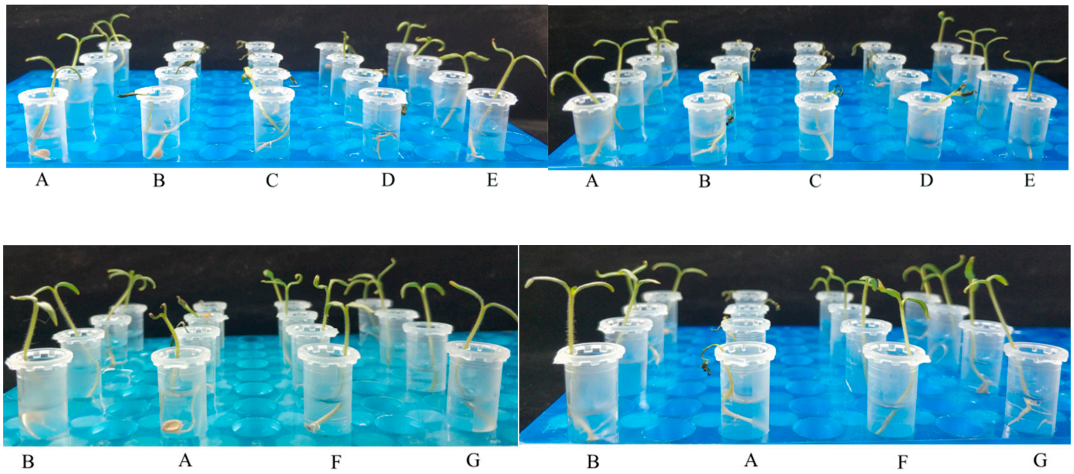

Figure S9. Control efficiency of strain NEAU-HV9 against *R. solanacearum*. A, tomato seedlings were inoculated with sterile water (CK 1); B, tomato seedlings only were inoculated with *R. solanacearum* (CK 2); C, tomato seedlings were preinoculated with suspension (10^7 cfu mL^{-1}) of NEAU-HV9 and then inoculated with *R. solanacearum* (TR 1); D, tomato seedlings were preinoculated with suspension (10^8 cfu mL^{-1}) of NEAU-HV9 and then inoculated with *R. solanacearum* (TR 1); E, tomato seedlings were preinoculated with suspension (10^9 cfu mL^{-1}) of NEAU-HV9 and then inoculated with *R. solanacearum* (TR 1); F, actinomycin D at the concentration 1 × MIC (TR 2); G, actinomycin D at the concentration 2 × MIC (TR 2).

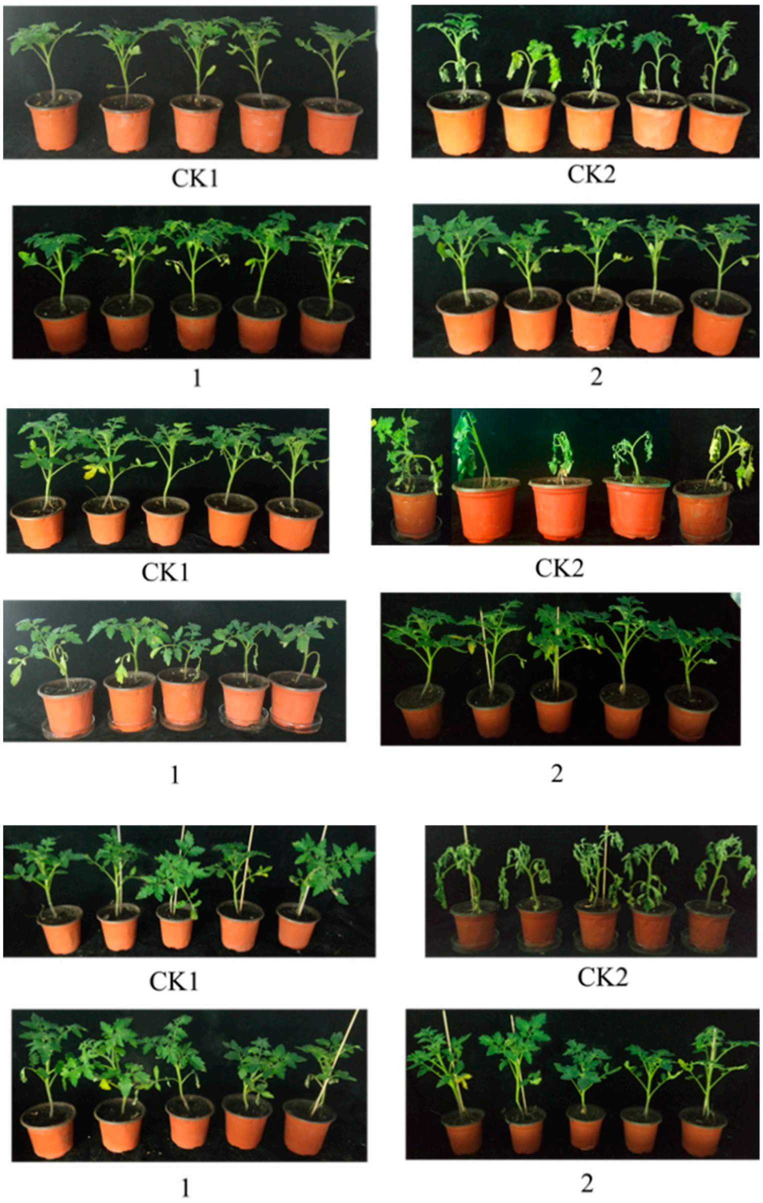

Figure S10. Control efficiency of the actinomycin D and strain NEAU-HV9 against *R. solanacearum*. CK 1, positive control; CK 2, negative control; 1, tomato seedlings were preinoculated with suspension (10^9 cfu g^{-1}) of NEAU-HV9; 2, actinomycin D at the concentration 1 × MIC.
